# Primary mental healthcare for adults with mild intellectual disabilities: a Dutch database study

**DOI:** 10.1080/13814788.2022.2142936

**Published:** 2022-11-22

**Authors:** Katrien P. M. Pouls, Monique C. J. Koks-Leensen, Willem J. J. Assendelft, Mathilde Mastebroek, Geraline L. Leusink

**Affiliations:** Department of Primary and Community Care, Radboud University Medical Center, Nijmegen, the Netherlands

**Keywords:** Primary healthcare, general practitioners, intellectual disability, mental health, medical record

## Abstract

**Background:**

General practitioners (GPs) are increasingly confronted with people with both mild intellectual disability (MID) and mental health (MH) problems. Little is known about the type of MH problems for which people with MID visit their GP and the care provided.

**Objectives:**

To identify the type and prevalence of MH disorders and MH-related complaints in people with MID in primary care and care provided, compared to people without ID.

**Methods:**

By linking the Netherlands Institute for Health Services Research’s primary care databases, comprising electronic health records, with Statistic Netherlands’ social services and chronic care databases, we identified 11,887 people with MID. In this four-year retrospective study, MH-related International Classification of Primary Care (ICPC) codes and care characteristics were compared between people with MID and without ID.

**Results:**

Of the people with MID, 48.8% had MH problems recorded vs. 30.4% of the people without ID, with significant differences in substance abuse, suicide attempts, and psychosis. Of the MID group, 80.3% were not registered by their GP with the ICPC code mental retardation. GPs provided more care to people with MID and MH problems than people without ID but with MH-problems regarding consultations (median 6.4 vs. 4.0 per year) and variety of prescribed medications (median 2.7 vs. 2.0 per year).

**Conclusion:**

In primary care, the prevalence of MH problems and care provided is high in people with MID. To improve primary mental healthcare for this group, it is essential to increase GPs’ awareness and knowledge on the combination of MID and MH.

KEY MESSAGES

- Nearly half of patients with mild intellectual disability (MID) experience mental health (MH) problems.
- Patients with both MID and MH problems are provided with more consultations and medication prescriptions than patients without intellectual disability (ID) or with MID alone.
- In more than 80% of the patients with MID, the ID was not properly registered and potentially unrecognised by the GP.

## Introduction

People with mild intellectual disability (MID), characterised by a significant deficit in intellectual and adaptive functioning [1], suffer from more mental health (MH) disorders compared to people without intellectual disability (ID) but often do not receive appropriate mental healthcare [2–8]. General practitioners (GPs) are often the first point of contact for people with MH problems and are gatekeepers to specialised mental healthcare services [9]. Prevalence studies in primary care with a specific focus on mental health in people with MID are scarce and focus on established MH disorders only, implicating a lack of knowledge on MH-related complaints (problems presented, no established diagnoses) in primary care.

There are several reasons for concern. First, the identification of both MH disorders and MID are problematic [2,9,10]. Second, people with ID experience general health disparities, including mental health, because of barriers to providing timely, appropriate, and effective primary healthcare [6]. Long-term conditions, like psychosis and depression, are poorly managed in primary care and psychotropic prescriptions exceed the number of reported MH disorders, suggesting inappropriate prescriptions [2,5,9]. Finally, GPs themselves indicate a lack of knowledge and feel insecure about providing the care needed [9,11].

Nevertheless, little is known about the type of MH problems for which people with MID visit their GP and the care they receive. This primary care database study aims to provide an overview of the prevalence of both MH disorders and MH complaints in people with MID and the care provided in terms of consultations and medication prescriptions compared to people without ID. In addition, we study how often the GP adequately codes a person with MID.

## Method

### Study design

For this retrospective database study, we used databases from the Netherlands Institute for Health Services Research Primary Care Database (NIVEL-PCD) and Statistic Netherlands (SN) [12,13]. NIVEL collects healthcare data from routine electronic health register systems from over 400 representative Dutch general practices, covering approximately 10% of the Dutch population [12]. The NIVEL-PCD files provided data regarding personal characteristics, type of health problems, consultations, and medication prescriptions from 1 January 2015 to 31 December 2018. MH problems were identified by ICPC-P codes (International Classification of Primary Care, Psychological category) [14]. Medication prescriptions were coded according to the Anatomical Therapeutic Chemical (ATC) classification system up to level 3, the pharmacological subgroup [15]. We included persons 18 years or older in 2015 who remained in the NIVEL-PCD for > 1 year.

### Selection of study objectives

**Figure 1.**
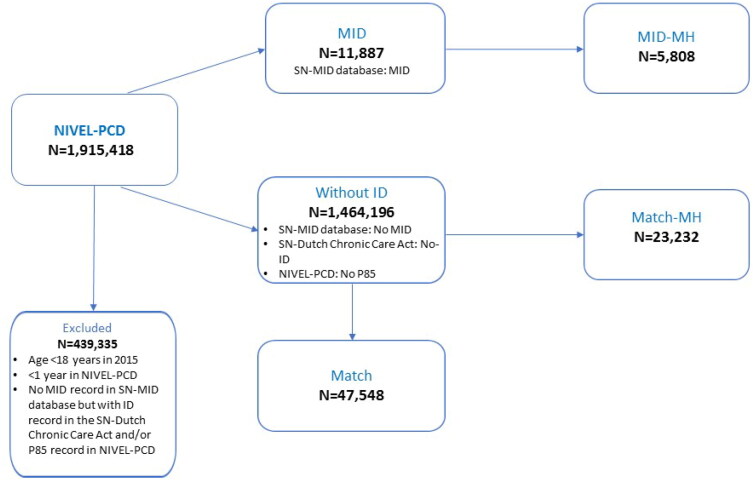
Composition of groups. NIVEL-PCD: Netherlands Institute for Health Services Research Primary Care Database; SN: Statistics Netherlands; MID: Group with mild intellectual disability; No-ID: Group with no-intellectual disability; MH: people who had one or more MH problems coded with an ICPC-P other than P85; Match: Match control group; MID-MH: Group with mild intellectual disability and a mental health problem; Match-MH: Match control group with MH problem.

To identify people with MID in the NIVEL-PCD, that database was linked with an SN-MID database. In this database, persons are identified who, in 2015, used services under the Dutch Chronic Care Act, the Disability Benefit Act, or the Sheltered Employment Act with a MID registration (Figure 1).

The comparison group without ID was not shown with a MID in the SN-MID database, had no ICPC code P85 (mental retardation) in the NIVEL-PCD, and was not eligible for ID-specific care according to the SN-Dutch Chronic Care Act database. We matched each individual from the MID group by age, sex and number of years in the dataset to four random individuals in the group without ID, to allow a robust comparison without overpowering.

We compiled a MID-MH group containing people who had one or more MH problems coded with an ICPC-P other than P85. An additional matched control group was asembled from the group without ID.

This study was reviewed and approved by the Medical Ethical Committee of Radboud University Medical Centre (2017-3921) and conducted according to the NIVEL and SN governance code. Results are reported per the RECORD statement.

### Outcomes

The MID was considered ‘registered’ by the GP if the person had a P85 code in the NIVEL-PCD. To calculate the period prevalence of MH problems, MH illness episodes were constructed according to Nielen et al.’s algorithm [16]. MH problems were divided into MH complaints with an ICPC code P1 to P29 and MH disorders with a code P70 to P99. We calculated the prevalence of unique MH problems and the median number of unique MH problems per patient during the research period. Additionally, ICPC codes relating to psychosis and substance abuse were combined and calculated per patient.

We described the number and type of consultations with a GP and mental health nurse practitioner (MHNP) and unique kinds of medication prescription categories. For comparison, we also described these for the MID group as a whole and their match group without ID, including people with and without MH problems. Each person’s medication prescription categories were recorded and divided into three selected categories: 1) Total medication use; 2) Psychotropics, including antipsychotics (N05A), anxiolytics (N05B), hypnotics and sedatives (N05C), antidepressants (N06A), psychostimulants (N06B), anti-dementia drugs (N06D), and drugs used in addictive disorders (N07B); and 3) Anti-epileptic medication (N03A), which may be prescribed for specific MH problems. It was impossible to link separate consultations and prescriptions to specific ICPC codes.

### Statistical analysis

Groups were stratified by age groups and sex. *Student’s t-*tests, Chi-squared tests, or Mann-Whitney *U*-tests were used to test statistical significance. Logistic regression was used to examine the association with the presence of MID on outcomes, calculating odds ratios (OR) with 95% confidence intervals (95% CI) adjusted for age, sex, and years registered in the database. A *p*-value < .05 was considered statistically significant. Only variables with a number of ≥ 30 people were included to ensure clinical relevance. All analyses were conducted using SPSS version 25.0.

## Results

### Demographics

In the NIVEL-PCD, 11,887 persons with MID were identified, with a mean age of 37.8 years, of whom 61.7% male, compared to, respectively, 48.4 years and 48.8% male in the group without ID (*n* = 1,464,196). Of the people with a MID, 80.3% had no ICPC code P85 registration (Table 1).

**Table 1.** Demographics.

		MID	Without ID
Total *N*		11,887	1,464,196
Men, *N* (%)		7338 (61.7)**^a^	714,136 (48.8)
Age, *M* (*SD*)		37.8 (15.0)**	49.4 (17.8)
Age groups, N (%)	18–24	3280 (27.6)**^b^	133,518 (9.1)
	25–34	2704 (22.7)	228,303 (15.6)
	35–44	1673 (14.1)	235,777 (16.1)
	45–54	2150 (18.1)	290,016 (19.8)
	55–64	1555 (13.1)	250,964 (17.1)
	65–74	525 (4.4)	325,618 (22.3)
Years registered in database, *M* (*SD*)		2.70 (1.15)*	2.76 (1.15)
ICPC P85 Mental retardation, *N* (%)		2339 (19.7)	n.a.

MID: Mild intellectual disability; ID: intellectual disability; ICPC: International Classification of Primary Care; **p < .001, ^a^MID compared with No-ID; ^b^age group distribution MID compared with No-ID.

### Period prevalence of MH problems

Of the people with MID, 48.9% had an MH problem over the four years period, either an MH complaint or an MH disorder, compared to 30.4% in the group without ID. They were significantly younger (37.1 vs. 50.6 years) and had a higher number of unique MH problems (median 2.0 vs. 1.0). In both groups, the prevalence of MH problems was higher in women. Table 2 presents the ICPC codes with the highest OR for both MH complaints and MH disorders (a full overview can be found in Supplementary Table A).

**Table 2.** Prevalence of mental health problems in persons with and without intellectual disability.

			MID *N* = 11,887	Without ID *N* = 1,464,196	OR (95%CI)^b^
MH problems (ICPC P1–P99)					
≥1 MH problem	N (%)	Total	5808 (48,9)	444,520 (30.4)	2.50 (2.41–2.59)**
		Men^a^	3.373 (46.0)	186,985 (26.2)	2.55 (2.43–2.67)**
		Women^a^	2435 (53.5)	257,535 (34.3)	2.43 (2.29–2.58)**
	Age		37.1 (14.3)**	50.6 (18.4)	
	Median unique MH problems (25–75 percentile)		2.0 (1.0–3.0)**	1.0 (1.0–3.0)	
MH complaint (ICPC P1–P29)					
≥1 MH complaint	*N* (%)		4299 (36.2)	313,200 (21.4)	2.39 (2.30–2.49)**
	Median unique MH complaints (25–75 percentile)		1.0 (1.0–2.0)**	1.0 (1.0–1.0)	
ICPC code^c^ *N* (%)	P28 Limited function/disability		303 (2.5)	1018 (0.1)	38.18 (33.34–43.72)**
	P24 Specific learning problem		116 (1.0)	1,424 (0.1)	6.23 (5.14–7.55)**
	P23 Adolescent behaviour symptom/complaint		96 (0.8)	2362 (0.2)	3.51 (2.86–4.32)**
	P04 Feeling/behaving irritably/angry		224 (1.9)	7809 (0.5)	3.47 (3.03–3.97)**
	P18 Medication abuse		101 (0.8)	5967 (0.4)	3.44 (2.82–4.20)**
	P15–P19 Substance abuse, any form		1,549 (13.0)	76,177 (5.2)	2.64 (2.50–2.79)**
MH disorders (ICPC P70–P99)					
≥1 MH disorders	N (%)		3,006 (25.3)	220,298 (15.0)	2.09 (2.00–2.18)**
	Median unique MH disorders (25–75 percentile)		1.0 (1.0–1.0)**	1.0 (1.0–1.0)	
ICPC code^c^ *N* (%)	P98 Psychosis NOS/other		242 (2.0)	5082 (0.3)	6.07 (5.32–6.92)**
	P71 Organic psychosis other		59 (0.5)	6858 (0.5)	4.43 (3.41–5.75)**
	P99 Psychological disorders, other		566 (4.8)	14,310 (1.0)	3.83 (3.51–4.18)**
	P77 Suicide/suicide attempt		110 (0.9)	3318 (0.2)	3.80 (3.14–4.60)**
	P72 Schizophrenia		158 (1.3)	5348 (0.4)	3.36 (2.87–3.95)**
	P71–73 and/or P98 Psychosis, any form		500 (4.2)	20,645 (1.4)	4.37 (3.99–4.79)**

MID: mild intellectual disability; ID: intellectual disability; MH: Mental health; ICPC: International Classification of Primary Care.

**p < .001, MID compared with No-ID; ^a^percentage of the total number of men/women within the group; ^b^OR adjusted for age, sex, and years registered in the database; ^c^Overview of the 5 ICPC codes of MH complaints/disorders with the highest odds and combined related codes.

More people with MID experienced an MH complaint than those without ID (36.2 vs. 21.4%; *p* < .001). The largest differences were seen in the codes ‘limited function and disability’ (P28; OR 38.18, 95% CI 33.34–43.72), ‘specific learning problems’ (P24; OR 6.23, 95% CI 5.14–7.55). In addition, 13.0% of the people with MID had an ICPC code associated with substance abuse (P15–P19), compared to 5.2% in the group without ID.

The prevalence of MH disorders was also higher in people with MID than those without ID (25.3 vs. 15.0%; *p* < .001). Large differences were seen in the codes ‘suicide/suicide attempt’ (P77; OR 3.80, 95% CI 3.14–4.60) and ‘psychological disorders, others’ (P99; OR 3.83, 95% CI 3.51–4.18). In addition, 4.2% of the people with MID had an ICPC code associated with psychosis (P71–73 and/or P98), compared to 1.4% in the group without ID.

### Care provided

The GP had a median of 6.4 consultations per person per year in the MID group with an MH problem, compared to 4.0 consultations in the matched group without ID (Table 3). In the MID group as a whole, this was 4.3 consultations versus 2.3 in the group without ID (Supplementary Table B). In all groups, women had more consultations. Slightly fewer people in the MID group with MH problems had an MHNP consultation (24.8 vs. 26.7%; *p* <.001), and both the GP and the MHNP provided more short than lengthy consultations and more home visits to people with both MID and MH problems, compared to the matched group without ID (Table 3).

**Table 3.** Consultations with general practitioners and mental health nurse practitioners.

			MID-MH *N* = 5808	Match-MH^b^ *N* = 32,232	OR (95%CI)^c^
GP consultations					
People with ≥1 consultation during research period, *N* (%)		Total	5702 (98.2)	22,708 (97.7)	1.29 (1.04–1.60)**
		Men^a^	3283 (97.3)	13,070 (96.9)	1.22 (0.97–1.54)
		Women^a^	2419 (99.3)	9638 (99.0)	1.67 (0.98–2.83)
Median consultations per year (25–75 percentile)		Total	6.4 (3.3–11.5)**	4.0 (2.0–7.3)	
		Men	5.0 (2.5–8.8)**	3.3 (1.7–5.8)	
		Women	9.0 (5.0–15.2)**	5.5 (3.0–9.0)	
Type of consultation^x^	Total (*N*)		141,267	357,841	
	Short consultation, *N* (%)		66,173 (46.8)	163,943 (45.8)	1.05 (1.03–1.06)**
	Long consultation, *N* (%)		21,052 (14.9)	60,504 (16.9)	0.86 (0.85–0.88)**
	Home visit short, *N* (%)		1626 (1.2)	2002 (0.6)	2.12 (1.98–2.26)**
	Home visit long, *N* (%)		1255 (0.9)	2571 (0.7)	1.25 (1.16–1.33)**
	Consultation by phone*, N* (%)		50,680 (35.9)	125,560 (35.1)	1.03 (1.02–1.05)**
	E-mail consultation, *N* (%)		481 (0.3)	3261 (0.9)	0.37 (0.34–0.41)**
MHNP consultations					
People with ≥1 consultation during research period, *N* (%)		Total	1442 (24.8)	6195 (26.7)	0.91 (0.85–0.98)*
		Men^a^	710 (21.0)	3130 (23.2)	0.89 (0.81–0.97)*
		Women^a^	732 (30.1)	3065 (31.5)	0.94 (0.85–1.04)
Median consultation per year (25–75 percentile) in people ≥1 consultation in research period		Total	1.5 (0.8–3.3)	1.3 (0.7–2.7)	
		Men	1.3 (0.7–2.8)	1.3 (0.7–2.4)	
		Women	1.5 (0.8–4.0)	1.5 (0.8–3.0)	
Type of consultation^d^	Total (*N*)		11,249	36,125	
	Short consultation, *N* (%)		274 (2.4)	564 (1.6)	1.62 (1.40–1.88)**
	Long consultation, *N* (%)		8586 (76.3)	30,711 (85.0)	0.57 (0.54–0.60)**
	Short home visit short, *N* (%)		15 (0.1)	17 (0.0)	not applicable
	Long home visit, *N* (%)		288 (2.6)	204 (0.6)	4.16 (3.46–5.00)**
	Consultation by phone, *N* (%)		1994 (17.7)	3949 (10.9)	1.77 (1.67–1.88)**
	E-mail consultation, *N* (%)		91 (0.8)	572 (1.6)	0.53 (0.42–0.66)**
	Group consultations, *N* (%)		<10^e^	108 (0.3)	not applicable

MID-MH: Group with mild intellectual disability and a mental health problem; Match-MH: Match control group with MH problem; GP: General practitioner; MHNP: Mental health nurse practitioner; **p < .001, *p < .05, MID compared with No-ID; ^a^percentage of the total number of men/women within the group; ^b^Match-MH: each individual from the MID-MH group randomly matched by age, sex, and number of years in the dataset to four individuals in the No-ID group; ^c^OR calculated for variable with an absolute number of ≥ 30 people in one of the groups, adjusted for age, sex, and years registered in the database; ^d^Long = >20 min, short = <20 min, percentage of total number of consultations; ^e^Absolute numbers below 10 may are not displayed.

The GP provided a median of 2.7 unique types of medication prescription categories per person per year in the MID group with an MH problem, compared to 2.0 prescriptions in the matched group without ID (Table 4). In the whole MID group, this was 2.0 prescriptions vs. 1.0 in the group without ID (Supplementary Table B). Of the MID group with an MH problem, 55.4% received at least one psychotropic prescription (OR 1.39, 95% CI 1.31–1.47), 19.9% an antipsychotic (N05A; OR 3.15, 95% CI 2.90–3.41), and 24.9% an anxiolytic (N05B; OR 1.41, 95%CI 1.32–1.51). In addition, 8.7% received an anti-epileptic prescription (N03A; OR 2.05, 95%CI 1.83–2.29).

**Table 4.** Medication prescriptions.

	MID-MH *N =* 5808	Match-MH *N =* 23,232	OR (95%CI)^a^
All medication			
≥1 prescription during research period, *N* (%)	5450 (93.8)	21,452 (92.3)	1.31 (1.16–1.48)**
Median types prescriptions per year (25–75 percentile)	2.7 (1.3–4.5)**	2.0 (1.0–3.3)	
Psychotropics			
≥1 prescription during research period, *N* (%)	3271 (55.4)	11,072 (47.7)	1.39 (1.31–1.47)**
Median types prescriptions per year in people ≥1 psychotropic prescription in research period (25–75 percentile)	0.5 (0.3–1.0)**	0.5 (0.3–1.0)	
N05A Antipsychotics, *N* (%)	1158 (19.9)	1716 (7.3)	3.15 (2.90–3.41)**
N05B Anxiolytics, *N* (%)	1,446 (24.9)	4473 (19.3)	1.41 (1.32–1.51)**
N05C Hypnotics and sedatives, *N* (%)	949 (16.3)	3603 (15.5)	1.07 (0.99–1.16)
N06A Antidepressant, *N* (%)	1,459 (25.1)	5,096 (21.9)	1.20 (1.12–1.29)**
N06B Psychostimulants, *N* (%)	275 (4.7)	1177 (5.1)	0.93 (0.81–1.07)
N06D Anti-dementia drugs, *N* (%)	<10^b^	23 (0.1)	not applicable
N07B Drugs used in addictive disorders, *N* (%)	385 (6.6)	1150 (5.0)	1.38 (1.22–1.55)**
Anti-epileptics			
≥1 anti-epileptic N03A prescription during research period, *N* (%)	504 (8.7)	1048 (4.5)	2.05 (1.83–2.29)**

MID-MH: Group with mild intellectual disability and a mental health problem; Match-MH: Match control group with MH problem.

**p < .001, MID compared with No-ID; ^a^OR calculated for variable with an absolute number of ≥ 30 people in one of the groups, adjusted for age, sex, and years registered in the database; ^b^Absolute numbers below 10 may are not displayed.

## Discussion

### Main findings

In this primary care database study, we found that almost half of the people with MID experienced MH problems versus one-third of those without ID, with large differences in substance abuse, suicide attempts, and psychosis. GPs provided more consultations and types of medication prescriptions to people with a combination of MID and an MH problem compared to matched people without ID or with MID alone. More than 80% of the persons with MID were not registered by the GP as such.

### Strengths and limitations

This study’s unique focus on people with mild intellectual disabilities is an important strength. We identified people with MID in primary care by linking a primary care database with a social services information database. This fills a blind spot about people with MID who would have been overlooked without data linkage. Another important strength is that this study does not focus only on MH disorders but gives a broader view by including MH complaints.

There are some limitations regarding databases containing routinely collected (health) data. In the NIVEL-PCD, details of diagnoses and treatments were limited and the care provided could not be linked to specific ICPC-P codes. The SN-MID database is composed mainly of users of work-related social services, resulting in an under-representation of older people. Finally, the database contains no exact information on intellectual and adaptive functioning on an individual level, so it cannot be ruled out that some people with more severe ID or borderline intelligence were included in our MID group. However, the SN-MID database is currently the most complete method available in the Netherlands to identify people with MID.

### Comparison with existing literature

In our study, 25.3% of the people with MID had an MH disorder. This is comparable to Sheehan et al.’s primary care cohort study [2], which found a point prevalence of 21% in people with any form of ID. Our incorporation of MH complaints has not been studied before in primary care.

The high prevalence of psychosis in MID (4.2%) we found was also observed in other primary care studies [5,17]. We found a higher prevalence of substance abuse in people with MID than those without ID. A systematic review that did not include primary care settings concluded that people with MID are at high risk of developing substance use disorder [18]. The striking relatively high prevalence of ‘suicide/suicide attempt’ (P77) and unspecified ICPC-P codes (P29, P99) has not been previously reported in primary care studies. However, Dodd et al.’s systematic review of suicidality in people with ID revealed that having an MH disorder is an important risk factor for suicide in these patients [19]. More importantly, it shows a lack of well-designed studies on suicide in people with ID, and there are limited ID-specific assessments or therapeutic interventions for suicidality.

The absence of a P85 code for most people with MID is in line with previous research [10]. The relatively high prevalence of the ICPC codes ‘limited function/disability’ (P28) and ‘specific learning problems’ (P24) in people with MID in this study could indicate that these codes are used as a substitute for P85.

The higher number of GP consultations, home visits, and telephone consultations for people with MID and MH problems is in line with earlier research on people with ID in general in primary care [17,20,21]. The relatively high number of medication prescriptions, especially psychotropics, for people with MID is consistent with earlier primary care research concerning people with ID [2,20,22]. From earlier research it is known that people with ID have greater healthcare needs with higher morbidity and premature mortality levels than patients without ID, a situation to which insufficient quality of healthcare is a substantial contributor [23]. Therefore, the differences that we found in the prevalence of MH problems and provided care between patients with MID and without ID indicate different healthcare needs in this specific patient group. However, our recent review on primary MH care to people with ID revealed that current primary MH care to this patient group is of an insufficient standard in terms of underdiagnosis of MH disorders, overmedication, and lack of effective patient follow-up, as well as limited GP experience in managing these patients [9]. A possible reflection of the reported difficulties in providing adequate MH care to these patients may contribute to the differences observed.

### Implications for research and practice

Timely recognition and treatment of not only MH disorders but also MH complaints are essential for the physical and emotional well-being and thus for the quality of life, of people with MID and therefore needs to be prioritised [24]. This requires GPs to be aware of the high prevalence of MH problems in people with MID and to be aware and knowledgeable about the effect of MID on symptom presentation, communication, and treatment. This study’s results give rise to several opportunities to improve the quality of primary MH care for people with MID. First, the relatively high use of unspecified ICPC-P codes (P29, P99) and the relatively high number of people with MID who received psychotropic prescriptions could be signs that GPs experience difficulties in classifying and treating MH problems in people with MID. Communication difficulties, an atypical presentation of MH symptoms, and diagnostic masking or overshadowing, where symptoms are obscured by the ID or mislabelled, can contribute to these difficulties [25]. Therefore, research on applicable primary MH guidelines for this patient group is important. Second, it is highly recommended to invest in recurring specific (postgraduate) training programmes for GPs. The relatively high prevalence of substance abuse and suicide in our study suggests that a proactive and preventive approach, aimed at identifying risk factors for MH problems and providing health education, should be part of this education. Third, GPs should be aware of the importance of identifying and registering MID for good care provision and for research purposes. ID screening tools can help identify MID and have been developed for GP practice but further implementation is needed [26,27]. GPs should reach clear agreement about when and how MID is recorded.

Fourth, it is important that primary care is accessible for people with MID and that their (mental) health needs are met. Future research should address the nature of the identified high-care utilisation in more detail. Our findings may be seen as a sign of additional healthcare needs and more intensive GP care provided to this patient group, putting a relatively high demand on primary care practices. As previous research indicated, GPs do not feel confident about providing care to people with ID [28], particularly those with additional MH problems [9,11]. Support for GPs may help them better address these complex health needs and improve the care that they provide. According to patients with ID, adaptations in how care is provided could be helpful, such as enhancing GPs’ communication skills, extra consultation time, continuity of care from the same GP, and involvement of family or carers in consultations and information provision [29,30]. Periodic health assessments, supported by an instrument, are another way to identify (mental) health needs [31]. Finally, although our database contained no information on referrals and consultations, GPs need to look to care professionals in their direct network for collaboration and support. Effective collaboration, specifically, is regarded as beneficial for the outcome of mental healthcare in primary care [32]. Therefore it is important to learn from best practices [32–34] and invest in (local) integrative and collaborative primary MH care models.

It remains of utmost importance to actively engage GPs and patients with MID in developing the suggested improvement strategies to ensure that they meet their needs and are applicable and feasible in daily practice.

## Conclusion

We found that MID is most often not registered by GPs and most likely partially unrecognised. Almost half of the people with MID visited the GP with an MH problem and were provided with more care in consultations and types of prescribed medication categories than those without ID or with MID alone. This may indicate that people with both MID and MH problems have even higher healthcare needs than people with MID alone, with a corresponding higher demand for primary care. In addition, our results suggest that GPs struggle to register the MID as such, to establish the correct MH diagnosis and, consequently, to provide appropriate treatment. These findings illustrate the relevance of improving the quality of primary MH care for people with MID. This may be achieved by creating more GP awareness and knowledge of MH problems in people with MID, the importance of MID recognition, additional (care) needs, and the need for collaboration with relevant local care professionals.

## Supplementary Material

Supplemental Tables

## Data Availability

Aggregated data from the databases used in this study are publicly available on a dedicated website of Statistics Netherlands (http://statline.cbs.nl), and Netherlands Institute for Health Services Research (https://www.nivel.nl/en/nivel-zorgregistraties-eerste-lijn/nivel-primary-care-database). The non-public microdata used to link databases is, under certain conditions, accessible for statistical and scientific research (fees apply). Procedures can be found at www.cbs.nl and www.nivel.nl. For further information: microdata@cbs.nl and zorgregistratie@nivel.nl
