# Supplementary material for: Primary mental healthcare for adults with mild intellectual disabilities: a Dutch database study
Source: Eur J Gen Pract. 2022 Nov 22;28(1):234–41. doi: 10.1080/13814788.2022.2142936 (PMC9683063; doi:10.1080/13814788.2022.2142936)
Supplement: Supplemental Tables [file IGEN_A_2142936_SM0855.docx]

| Supplementary Table A  Period prevalence of mental health problems | | | | | | |
| --- | --- | --- | --- | --- | --- | --- |
|  | | | | MID | Without ID | OR (95%CI)^c^ |
| Patients, N | | | **Total** | 11,887 | 1,464,196 |  |
|  |  |  | Men | 7,338 | 714,136 |  |
|  |  |  | Women | 4,549 | 750,060 |  |
| MH problems (P1–P99) | | | | | | |
| ≥1 MH problem | | N (%) | Total | 5,808 (48,9) | 444,520 (30.4) | 2.50 (2.41–2.59)^**^ |
|  |  |  | Men^a^ | 3.373 (46.0) | 186,985 (26.2) | 2.55 (2.43–2.67)^**^ |
|  |  |  | Women^a^ | 2,435 (53.5) | 257,535 (34.3) | 2.43 (2.29–2.58)^**^ |
|  |  | Age M(SD) | | 37.1 (14.3)^**^ | 50.6 (18.4) |  |
|  |  | Age group,  N (%^b^) | 18–24 | 1,600 (48.8) | 36,344 (27.2) | 2.75 (2.56–2.95)^**^ |
|  |  |  | 25–34 | 1,391 (51.4) | 66,434 (29.1) | 2.74 (2.54–2.96)^**^ |
|  |  |  | 35–44 | 881 (52.7) | 72,351 (30.7) | 2.67 (2.42–2.94)^**^ |
|  |  |  | 45–54 | 1,052 (48.9) | 88,513 (30.5) | 2.38 (2.18–2.59)^**^ |
|  |  |  | 55–64 | 697 (44.8) | 74,458 (29.7) | 2.04 (1.84–2.26)^**^ |
|  |  |  | 65–74 | 179 (36.7) | 51,500 (26.9) | 1.77 (1.47–2.13)^**^ |
|  |  |  | >75 | <10^d^ | 54,920 (40.9) | not applicable |
|  |  | Median number of unique MH problems (25–75 percentile) | | 2.0 (1.0–3.0)^**^ | 1.0 (1.0–3.0) |  |
| MH complaint (ICPC P1–P29) | | | | | | |
| ≥1 MH complaint | | N (%) | Total | 4,299 (36.2) | 313,200 (21.4) | 2.39 (2.30–2.49)^*^ |
|  |  |  | Men^a^ | 2,541 (34.6) | 136,786 (19.2) | 2.40 (2.29–2.52)^*^ |
|  |  |  | Women^a^ | 1,758 (38.6) | 176,414 (23.5) | 2.37 (2.23–2.52)^*^ |
|  |  | Median number of unique MH complaints (25–75 percentile) | | 1.0 (1.0–2.0)^**^ | 1.0 (1.0–1.0) |  |
| ICPC code  N (%) | P01 Feeling anxious/nervous/tense | | | 573 (4.8) | 50,158 (3.4) | 1.61 (1.48–1.76)^**^ |
|  | P02 Acute stress reaction | | | 648 (5.5) | 36,848 (2.5) | 2.16 (1.99–2.34)^**^ |
|  | P03 Feeling depressed | | | 372 (3.1) | 35,104 (2.4) | 1.31 (1.18–1.46)^**^ |
|  | P04 Feeling/behaving irritably/angry | | | 224 (1.9) | 7,809 (0.5) | 3.47 (3.03–3.97)^**^ |
|  | P05 Senility, feeling/behaving old | | | <10^d^ | 3,759 (0.3) | not applicable |
|  | P06 Sleep disturbance | | | 829 (7.0) | 84,341 (5.8) | 1.72 (1.60–1.85)^**^ |
|  | P07 Sexual desire reduced | | | 16 (0.1) | 1,849 (0.1) | not applicable |
|  | P08 Sexual fulfilment reduced | | | 56 (0.5) | 4,543 (0.3) | 1.40 (1.08–1.83)^*^ |
|  | P09 Sexual preference concern | | | 21 (0.2) | 597 (0.0) | not applicable |
|  | P10 Stammering/stuttering/tic | | | 21 (0.2) | 552 (0.0) | not applicable |
|  | P11 Eating problem in child | | | <10 | 123 (0.0) | not applicable |
|  | P12 Bedwetting/enuresis | | | 27 (0.2) | 434 (0.0) | not applicable |
|  | P13 Encopresis/bowel training problem | | | <10^d^ | 50 (0.0) | not applicable |
|  | P15 Chronic alcohol abuse | | | 364 (3.1) | 15,062 (1.0) | 3.12 (2.80–3.47)^**^ |
|  | P16 Acute alcohol abuse | | | 81 (0.7) | 2,489 (0.2) | 3.03 (2.43–3.79)^**^ |
|  | P17 Tobacco abuse | | | 799 (6.7) | 49,034 (3.3) | 2.09 (1.95–2.25)^**^ |
|  | P18 Medication abuse | | | 101 (0.8) | 5,967 (0.4) | 3.44 (2.82–4.20)^**^ |
|  | P19 Drug abuse | | | 407 (3.4) | 9,233 (0.6) | 3.23 (2.91–3.57)^**^ |
|  | P20 Memory disturbance | | | 140 (1.2) | 28,352 (1.9) | 1.59 (1.34–1.88)^**^ |
|  | P21 Overactive/hyperkinetic syndrome | | | 367 (3.1) | 12,463 (0.9) | 2.09 (1.88–2.33)^**^ |
|  | P22 Child behaviour symptom/complaint | | | 99 (0.8) | 9,408 (0.6) | 1.33 (1.09–1.62)^*^ |
|  | P23 Adolescent behaviour symptom/complaint | | | 96 (0.8) | 2,362 (0.2) | 3.51 (2.86–4.32)^**^ |
|  | P24 Specific learning problem | | | 116 (1.0) | 1,424 (0.1) | 6.23 (5.14–7.55)^**^ |
|  | P25 Phase of life problem adult | | | 16 (0.1) | 4,137 (0.3) | not applicable |
|  | P27 Fear of mental disorder | | | <10^d^ | 822 (0.1) | not applicable |
|  | P28 Limited function/disability | | | 303 (2.5) | 1,018 (0.1) | 38.18 (33.34–43.72)^**^ |
|  | P29 Psychological symptom/complaints other | | | 459 (3.9) | 25,528 (1.7) | 2.07 (1.89–2.28)^**^ |
|  | P15–19 Substance abuse, any form | | | 1,549 (13.0) | 76,177 (5.2) | 2.64 (2.50–2.79)^**^ |
| MH disorders (ICPC P70-P99) | | | | | | |
| ≥1 MH disorders | | N (%) | **Total** | 3,006 (25.3) | 220,298 (15.0) | 2.09 (2.00–2.18) |
|  |  |  | Men^a^ | 1,646 (22.4) | 84,198 (11.8) | 2.22 (2.10–2.35) |
|  |  |  | Women^a^ | 1,360 (29.9) | 136,100 (18.1) | 1.92 (1.82–2.07) |
|  | | Median number of unique MH disorders (25–75 percentile) | | 1.0 (1.0–1.0)^**^ | 1.0 (1.0–10) |  |
| ICPC code, N (%) | P70 Dementia | | | 24 (0.2) | 15,512 (1.1) | not applicable |
|  | P71 Organic psychosis other | | | 59 (0.5) | 6,858 (0.5) | 4.43 (3.41–5.75)^**^ |
|  | P72 Schizophrenia | | | 158 (1.3) | 5,348 (0.4) | 3.36 (2.87–3.95)^**^ |
|  | P73 Affective psychosis | | | 87 (0.7) | 5,005 (0.3) | 2.49 (2.01–3.09)^**^ |
|  | P74 Anxiety disorder/anxiety state | | | 706 (5.9) | 54,668 (3.7) | 1.59 (1.48–1.72)^**^ |
|  | P75 Somatization disorder | | | 63 (0.5) | 4,004 (0.3) | 1.89 (1.47–2.43)^**^ |
|  | P76 Depressive disorder | | | 801 (6.7) | 76,663 (5.2) | 1.46 (1.36–1.58)^**^ |
|  | P77 Suicide/suicide attempt | | | 110 (0.9) | 3,318 (0.2) | 3.80 (3.14–4.60)^**^ |
|  | P78 Neuraesthenia/surmenage | | | 371 (3.1) | 47,088 (3.2) | 0.90 (0.82–1.00) |
|  | P79 Phobia/compulsive disorder | | | 86 (0.7) | 6,010 (0.4) | 1.56 (1.26–1.94)^**^ |
|  | P80 Personality disorder | | | 506 (4.3) | 22,623 (1.5) | 2.31 (2.11–2.53)^**^ |
|  | P98 Psychosis NOS/other | | | 242 (2.0) | 5,082 (0.3) | 6.07 (5.32–6.92)^**^ |
|  | P99 Psychological disorders, other | | | 566 (4.8) | 14,310 (1.0) | 3.83 (3.51–4.18)^**^ |
|  | P71–73 and/or P98 Psychosis, any form | | | 500 (4.2) | 20,645 (1.4) | 4.37 (3.99–4.79)^**^ |

MID = Mild intellectual disability; ID = Intellectual disability; MH = Mental health; ICPC = International Classification of Primary Care;

^**^p < .001 and ^*^p < .05 MID compared with No-ID; ^a^percentage of the total number of men/women within the group;

^b^percentage of the total number of persons in the age group within the group; ^c^OR calculated for variable with an absolute number of ≥ 30 people in one of the groups, adjusted for age, sex, and years registered in the database; Absolute numbers below 10 are not displayed.

| Supplementary Table B  Consultations and medication prescriptions | | | | | | | |
| --- | --- | --- | --- | --- | --- | --- | --- |
|  | | | | MID  N=11,887 | | Match No-ID  N=47,548 | OR (95%CI)^b^ |
| GP consultations | | | | | | | |
| People with ≥1 consultation during research period, N (%) | | **Total** | 10,967 (92.3) | | | 41,999 (88.3) | 1.66 (1.54–1.78)^**^ |
|  |  | Men^a^ | 6,575 (89.6) | | | 25,006 (85.2) | 1.56 (1.44–1.70)^**^ |
|  |  | Women^a^ | 4,392 (96.5) | | | 16,993 (93.3) | 2.06 (1.73–2.44)^**^ |
| Median number of consultations per registered year (25–75 percentile) | | **Total** | 4.3 (1.7–8.7)^**^ | | | 2.3 (1.0–4.8) |  |
|  |  | Men | 3.0 (1.0–6.5) | | | 1.8 (0.6–3.6) |  |
|  |  | Women | 6.7 (3.3–12.0) | | | 3.5 (1.6–6.3) |  |
| Type of consultation^c^ | Total (N) | | 206,381 | | | 457,777 |  |
|  | Short consultation, N (%) | | 102,758 (49.8) | | | 231,456 (50.6) | 0.94 (0.94–0.95)^**^ |
|  | Long consultations, N (%) | | 28,451 (13.8) | | | 66,544 (14.5) | 0.91 (0.90–0.93)^**^ |
|  | Home visit short, N (%) | | 2,315 (1.1) | | | 2,890 (0.6) | 2.16 (2.08–2.26)^**^ |
|  | Home visit long, N (%) | | 1,830 (0.9) | | | 3,070 (0.7) | 1.47 (1.40–1.54)^**^ |
|  | Consultations by phone, N (%) | | 70,349 (34.1) | | | 150,016 (32.8) | 1.07 (1.06–1.08)^**^ |
|  | E-mail consultations, N (%) | | 678 (0.3) | | | 3,801 (0.8) | 0.38 (0.35–0.41)^**^ |
| Total prescriptions of medication | | | | | | | |
| People with ≥1 prescription during research period, N (%) | | | 10,362 (87.2) | | 38,271 (80.5) | | 1.76 (1.66–1.87)^**^ |
| Median type prescriptions per year (25–75 percentile) | | | 2.0 (0.8–3.8)^**^ | | 1.0 (0.3–2.5) | |  |

MID = Group with mild intellectual disability; Match No-ID = Match control group with no intellectual disability; GP = General practitioner;

^**^p < .001 MID compared with No-ID; ^a^percentage of the total number of men/women in the group; ^b^ OR calculated for variable with an absolute number of ≥ 30 people in one of the groups, adjusted for age, sex, and years registered in the database; ^c^Long = >20 minutes, short = <20minutes, percentage of total number of consultations
